# Short-Coupled Purkinje Ventricular Fibrillation Triggers Successfully Suppressed With Flecainide

**DOI:** 10.1016/j.jaccas.2026.108863

**Published:** 2026-06-15

**Authors:** Ela Ahmad, Mohamed H. Abdalaziz, Adam Tawney, Nishaki Mehta, Ilana B. Kutinsky

**Affiliations:** a Department of Cardiovascular Medicine, Case Western Reserve University MetroHealth System, Cleveland, Ohio, USA; b Department of Internal Medicine, Corewell Health William Beaumont University Hospital, Royal Oak, Michigan, USA; c Department of Cardiovascular Medicine, Corewell Health William Beaumont University Hospital, Royal Oak, Michigan, USA

**Keywords:** flecainide, idiopathic ventricular fibrillation, sudden cardiac arrest

## Abstract

**Background:**

Idiopathic ventricular fibrillation (IVF) affects young individuals without identifiable structural or channelopathic disease. Short-coupled premature ventricular contractions (sc-PVCs) of Purkinje origin are a recognized trigger, yet optimal pharmacotherapy remains undefined.

**Case Summary:**

A 28-year-old healthy woman presented after a witnessed out-of-hospital sudden cardiac arrest. Extensive workup excluded all structural, ischemic, and inherited causes. Telemetry identified sc-PVCs of left anterior fascicle Purkinje origin, with a coupling interval of 330 ms, reproducibly triggering ventricular fibrillation. Intravenous amiodarone and esmolol failed, while flecainide achieved complete sc-PVC suppression. A subcutaneous implantable cardioverter-defibrillator was implanted.

**Discussion:**

IVF recurs in 25% to 40% of patients, mandating implantable cardioverter-defibrillator placement and trigger suppression. Quinidine holds an 83.3% efficacy rate but is limited by side effects and availability. Flecainide achieved comparable suppression with sustained benefit at 12-month follow-up on monotherapy.

**Take-Home Message:**

Flecainide is a promising alternative to quinidine for suppressing sc-PVC-triggered IVF, offering comparable efficacy and better tolerability.

## Visual Summary

**Figure:**
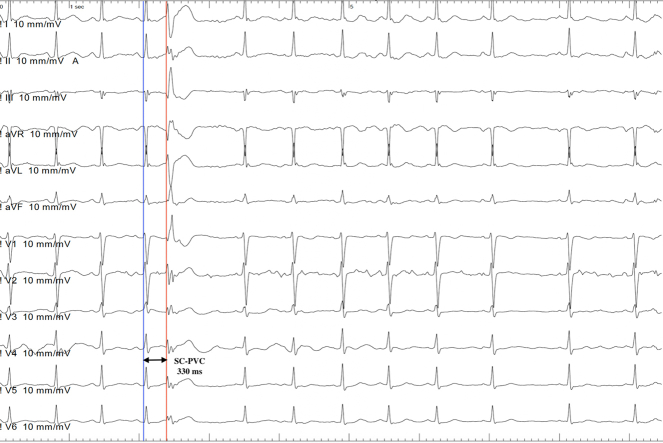

## History of Presentation

A 28-year-old white female presented to the hospital following a witnessed sudden cardiac arrest. She was at her workplace when she collapsed from a chair and fell to the ground. Immediate cardiopulmonary resuscitation was initiated by her coworkers. Emergency medical services arrived within 3 minutes and found the patient pulseless, apneic, and unresponsive. Initial rhythm analysis was consistent with ventricular fibrillation (VF). She was defibrillated with 200 J followed by 2 minutes of CPR, leading to the return of spontaneous circulation. The patient remained unresponsive to commands following return of spontaneous circulation. Upon arrival to the emergency department, the patient was tachycardic with a heart rate of 171 beats/min, O_2_ saturation of 94% on 15 L/min via bag-valve mask, afebrile, and normotensive with a blood pressure of 125/75 mm Hg. On physical examination, she was markedly agitated. Cardiac examination revealed a tachycardic regular rhythm with no murmurs, rubs, or gallops on auscultation. Lungs were clear bilaterally, and no gross focal neurological deficits were noted. The patient was confused and unable to protect her airway, thus requiring endotracheal intubation. The overall clinical course and key diagnostic findings are illustrated in Figure 1.

**Figure 1:**
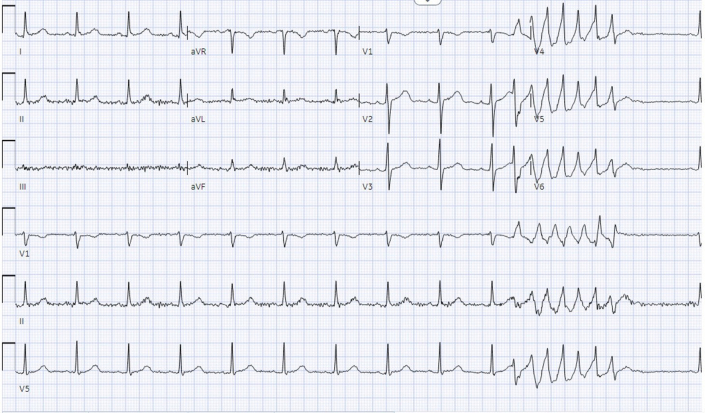
Presentation, Workup, Management, and Outcomes of a 28-Year-Old Woman With sc-PVC-Triggered Idiopathic VF Successfully Suppressed With Flecainide IVF = idiopathic ventricular fibrillation; LVEF = left ventricular ejection fraction; sc-PVCs = short-coupled premature ventricular contractions; S-ICD = subcutaneous implantable cardioverter-defibrillator.

## Past Medical History

No significant past medical history was noted.

## Differential Diagnosis

The differential diagnosis for sudden cardiac arrest is broad as it may be primarily of cardiac etiology (eg, acute myocardial infarction, massive pulmonary embolism, ventricular arrhythmia, cardiomyopathy, channelopathy, myocarditis, severe aortic stenosis, complete heart block, or congenital cardiac anomaly). Sudden cardiac arrest could also be secondary to a noncardiac etiology including intoxication/overdose, electrolyte abnormalities, cerebrovascular accident, intracranial hemorrhage, primary respiratory arrest, seizure, brain tumor, hypothermia, severe sepsis, or major trauma and blood loss.

## Investigations

Twelve-lead ECG showed sinus tachycardia, normal PR, QRS, and QT intervals, with no evidence of early repolarization as shown in Figure 2. In the setting of head trauma, computed tomography and computed tomography angiogram of head and neck were obtained and were within normal limits. The patient was then taken to the cardiac catheterization lab for an emergent coronary angiography which revealed normal coronaries. A pulmonary angiogram was also performed and was negative for pulmonary embolism. A 24-hour continuous electroencephalogram was consistent with severe bilateral cerebral dysfunction/encephalopathy, which is nonspecific with respect to underlying etiology but to which toxic-metabolic and systemic causes, acute or chronic central nervous system pathology, and/or medication-related effects may be contributory. No epileptiform activity was noted. A 2D Echo revealed a left ventricular ejection fraction of 60%, with no segmental wall motion abnormalities, a diastolic interventricular septum thickness of 5.2 mm, trace valvular regurgitation, and no pericardial effusion.

**Figure 2:**
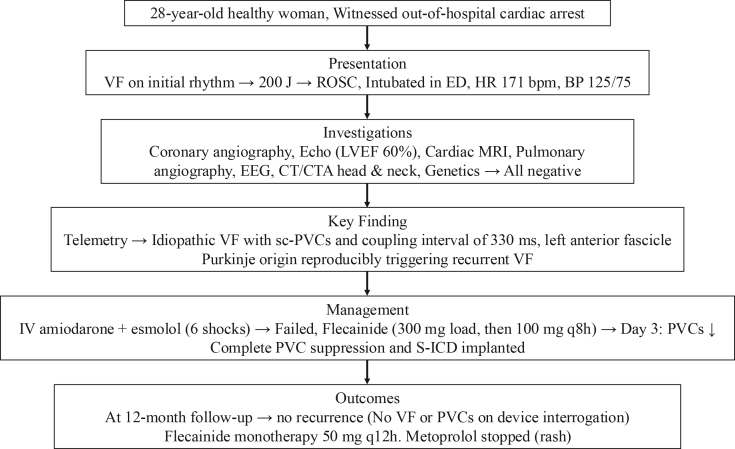
Sinus Tachycardia on Presentation BP = blood pressure; CT = computed tomography; CTA = computed tomography angiography; ED = emergency department; EEG = electroencephalogram; HR = heart rate; LVEF = left ventricle ejection fraction; MRI = magnetic resonance imaging; ROSC = return of spontaneous circulation; S-ICD = subcutaneous implantable cardioverter-defibrillator; sc-PVC = short-coupled premature ventricular contractions; VF = ventricular fibrillation.

Cardiac magnetic resonance showed normal cardiac size and function. There was no late gadolinium enhancement, and none of the Revised Padua Criteria for arrhythmogenic right ventricular cardiomyopathy were present. Telemetry monitoring showed frequent uniform short-coupled premature ventricular contractions (sc-PVCs) with a coupling interval of 330 ms. PVCs were narrow with a QRS duration of 100 ms and right bundle morphology. PVCs were felt to be consistent with a left anterior fascicle site of origin. The patient continued to have salvos of VF, which were clearly triggered by the same sc-PVCs. Early repolarization was later noted in inferior leads and leads I and aVL as demonstrated in Figures 3 and 4. Although they were not present on the presentation ECG, this suggests a diagnosis of early repolarization syndrome. However, this diagnosis is less likely, as early repolarization in these leads resolved on subsequent ECG despite the use of flecainide, which would have exacerbated the condition.

**Figure 3:**
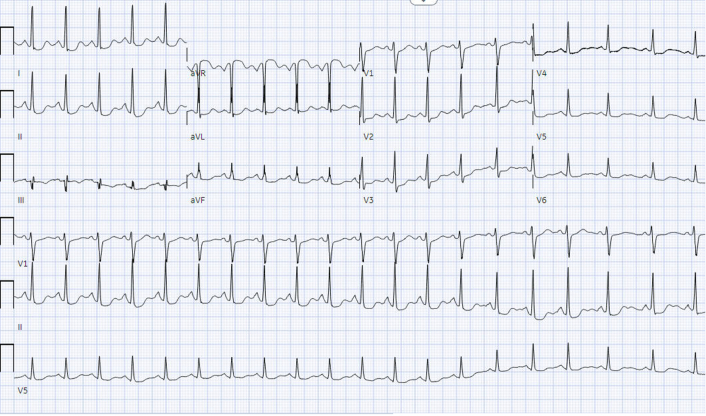
Narrow Short-Coupled Premature Ventricular Contractions, Morphology Consistent With Left Anterior Fascicle Site of Origin

**Figure 4:**
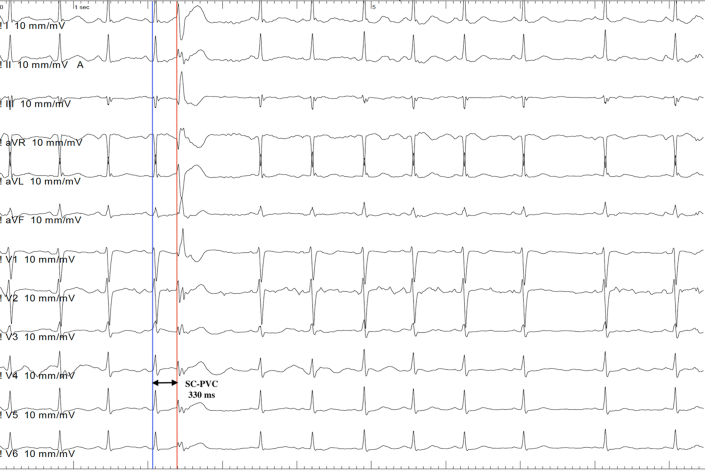
Self-Terminating Episode of Ventricular Fibrillation

## Management

The patient was started on intravenous (IV) amiodarone and esmolol; however, sc-PVCs persisted, triggering more episodes of VF. She received a total of 6 shocks with immediate defibrillation each time. Amiodarone was discontinued. Due to the suspicion of idiopathic VF (IVF), the patient was loaded with flecainide 300 mg, followed by flecainide 100 mg twice daily. Her sc-PVCs became infrequent and no longer triggered VF on day 3. Flecainide was then increased to 100 mg every 8 hours with complete suppression of sc-PVCs. A subcutaneous implantable cardioverter-defibrillator (ICD) was implanted for secondary prevention of VF. Defibrillation threshold (DFT) testing with serial 50-Hz inductions was attempted at implant yet failed to induce VF. Prior to discharge, IV esmolol was discontinued. The patient was started on oral metoprolol tartrate 50 mg every 6 hours, and the flecainide dose was reduced to 50 mg every 8 hours with no recurrence of sc-PVCs.

## Outcome and Follow-Up

Shortly after discharge, the patient reported a rash attributed to metoprolol tartrate, which was discontinued. She tolerated flecainide well and remained on 50 mg every 8 hours. At her 6-month follow-up visit, device interrogation revealed no recurrence of VF and no evidence of PVCs. Flecainide frequency was reduced to every 12 hours. At her 12-month follow-up visit, the patient continued to do well on the current dose with no recurrence to date. Genetic testing was performed and was unremarkable for arrhythmogenic genetic disorders. DFT testing was discussed in the outpatient setting; however, the patient declined further testing.

## Discussion

IVF is a challenging medical condition. Recurrence occurs frequently in 25% to 40% of patients within 2 years of index presentation.^1^ ICD placement is considered the first-line therapy for secondary prevention.^2^ However, a combination of pharmacotherapy and radiofrequency ablation in drug-refractory cases is frequently necessary to suppress triggers of arrhythmia. Clear guidelines on acute drug therapy for IVF are still lacking. A recent literature review with extended follow-up by Belhassen et al^3^ revealed that oral beta blockers were only effective in 13% of cases. Among the IV medications, phenytoin and quinidine were the most effective; however, the experience with these medications remains limited. IV verapamil had a 40% success rate while amiodarone and lidocaine success rates were 10% and 4.2%, respectively. Among the oral medications, quinidine was the most effective with a success rate of 83.3%, followed by amiodarone and flecainide (60% efficacy rate each) and verapamil (50%).^3^

In our patient, complete suppression of sc-PVCs was achieved with flecainide with no recurrence of VF. Our patient received a subcutaneous ICD prior to discharge. At implant, DFT testing with serial 50-Hz inductions failed to induce VF. A recently published subanalysis of the PRAETORIAN-DFT trial reports induction failure in 12 patients out of 482 who were randomized to the DFT arm, although only 45 patients (9.3% of patients in the DFT arm) had IVF.^4^ Different methods can be used for DFT testing including the step-down method, safety margin method, step-up method, and binary method; however, no single protocol showed superiority over the others.^5^ It is conceivable that failed induction of VF in our case is related to ongoing flecainide therapy at the time of the procedure. Our patient decided not to pursue any further DFT testing. She has also declined ablation. Quinidine is currently considered the first-line agent for suppression of sc-PVCs; however, given its side-effect profile and limited availability, flecainide may be a promising alternative given its comparable efficacy, fewer adverse effects, and lower doses required for arrhythmia suppression. Further research is warranted to determine its efficacy across a broader patient population.

## Conclusions

All IVF patients require ICD placement due to its frequent and unpredictable recurrence. Pharmacological suppression of IVF triggers, that is, sc-PVCs, is of paramount importance during the initial presentation. Catheter ablation of sc-PVCs triggering VF is effective but often performed at subsequent presentations due to ICD shocks or during the initial presentation if pharmacological suppression fails.

## Funding Support and Author Disclosures

The authors have reported that they have no relationships relevant to the contents of this paper to disclose.

**Take-Home Messages**

• Ventricular fibrillation treatment varies based on its underlying etiology.
• This case highlights the potential role of flecainide in suppressing short-coupled triggers of idiopathic ventricular fibrillation.
